# Development of plate-type and tubular chemiluminescence immunoassay against African swine fever virus p72

**DOI:** 10.1007/s00253-024-13249-5

**Published:** 2024-08-02

**Authors:** Chun Miao, Junjun Shao, Sicheng Yang, Shenghui Wen, Yunyun Ma, Shandian Gao, Huiyun Chang, Wei Liu

**Affiliations:** 1 State Key Laboratory for Animal Disease Control and Prevention, Lanzhou Veterinary Research Institute, Chinese Academy of Agricultural Sciences, Lanzhou, China; 2 Gansu Province Research Center for Basic Disciplines of Pathogen Biology, Lanzhou Veterinary Research Institute Chinese Academy of Agricultural Sciences, Lanzhou, China

**Keywords:** African swine fever virus, p72-CLIA, p72-MPCLIA, Diagnosis

## Abstract

**Abstract:**

African swine fever (ASF) is a highly contagious and fatal viral disease that has caused huge economic losses to the pig and related industries worldwide. At present, rapid, accurate, and sensitive laboratory detection technologies are important means of preventing and controlling ASF. However, because attenuated strains of African swine fever virus (ASFV) are constantly emerging, an ASFV antibody could be used more effectively to investigate the virus and control the disease on pig farms. The isolation of ASFV-specific antibodies is also essential for the diagnosis of ASF. Therefore, in this study, we developed two chemiluminescence immunoassays (CLIAs) to detect antibodies directed against ASFV p72: a traditional plate-type blocking CLIA (p72-CLIA) and an automatic tubular competitive CLIA based on magnetic particles (p72-MPCLIA). We compared the diagnostic performance of these two methods to provide a feasible new method for the effective prevention and control of ASF and the purification of ASFV. The cut-off value, diagnostic sensitivity (Dsn), and diagnostic specificity (Dsp) of p72-CLIA were 40%, 100%, and 99.6%, respectively, in known background serum, whereas those of p72-MPCLIA were 36%, 100%, and 99.6%, respectively. Thus, both methods show good Dsn, Dsp, and repeatability. However, when analytical sensitivity was evaluated, p72-MPCLIA was more sensitive than p72-CLIA or a commercial enzyme-linked immunosorbent assay. More importantly, p72-MPCLIA reduced the detection time to 15 min and allowed fully automated detection. In summary, p72-MPCLIA showed superior diagnostic performance and offered a new tool for detecting ASFV infections in the future.

**Key points:**

*• Two chemiluminescence immunoassay (plate-type CLIA and tubular CLIA) methods based on p72 monoclonal antibody (mAb) were developed to detect ASFV antibody.*

*• Both methods show good diagnostic performance (Dsn (100%), Dsp (99.6%), and good repeatability), and p72-MPCLIA detects antibodies against ASFV p72 with high efficiency in just 15 min.*

**Supplementary Information:**

The online version contains supplementary material available at 10.1007/s00253-024-13249-5.

## Introduction

African swine fever (ASF) was first identified in Kenya in 1921 and spread quickly to Europe. It reached Georgia in 2007 and thence the Russian Federation and Eastern Europe via the Caucasus). After nearly 10 years, ASF reached China in 2018, entailing huge economic losses, and it is still widespread throughout the world (Li et al. 2022b).

ASF is a deadly hemorrhagic disease caused by African swine fever virus (ASFV), affecting all types of pigs. Based on nucleotide variations in the C-terminal sequence of the p72 protein, encoded by the B646L gene, ASFV isolates can be classified into 24 genotypes (Malogolovkin et al. 2015). Genotypes I and II are currently prevalent in China (Gaudreault et al. 2020; Sun et al. 2021). As the sole member of the *Asfarviridae* family, ASFV possesses a large linear double-stranded DNA genome (Dixon et al. 2013). Its icosahedral structure, ~ 200 nm in diameter, comprises the inner core, inner core shell, inner membrane, outer capsid, and outer capsule membrane (Alejo et al. 2018; Andrés et al. 2001; Salas and Andrés, 2013). The ASFV genome encodes 68 structural proteins and 150–200 nonstructural proteins (Alejo et al. 2018; Dixon et al. 2013). The major capsid protein, p72, constitutes around 31–33% of the entire viral particle. Its strong immunogenicity and reactivity make it a suitable target protein for antibody detection and a widely used component in ASF diagnostic methods (Caixia et al. 2022; García-Escudero et al. 1998; Geng et al. 2022; Liu et al. 2019; Yu et al. 1996).

In recent years, the number of strains with high transmissibility and low virulence has increased significantly, reducing the accuracy of the methods used to detect ASFV nucleic acids (Sun et al. 2021). As the products of viral infections, antibodies can persist for months in the host or even the for life of the host, so methods based on antibodies greatly improve the accuracy of detection. Consequently, it is very important to develop a rapid, accurate, sensitive, and simple method for the detection of ASFV antibodies, to facilitate the large-scale screening of pigs on pig farms during the early stages of infection and the timely control of epidemic spread. Kollnberger et al. (Kollnberger et al. 2002) reported that p72 is one of the most immunogenic and suitable target proteins for the antibody-based detection of ASFV, and another study has shown that a detection method based on p72 is the most sensitive detection technology available at present (Wang et al. 2019), with good development prospects.

At present, there are many methods to detect ASFV, including PCR (Qi et al. 2023; Qiu et al. 2021) and ELISA (Liu et al. 2021a; Lv et al. 2021; Yu et al. 2021), which have shown good diagnostic performance. Among these, PCR, with its excellent sensitivity, specificity, and high-throughput capabilities, is considered the “gold standard” for early ASF diagnosis (Gallardo et al. 2015; Oura et al. 2013). To date, various PCR detection methods have been developed and validated, including conventional PCR, real-time quantitative PCR (qPCR) (Chen et al. 2021; Fernández-Pinero et al. 2013), and isothermal amplification analysis (Notomi et al. 2000). However, these PCR-based detection methods are not suitable for recovered or virus-carrying animals since their genomic detection levels are notably lower than the detection levels of PCR (Li et al. 2022b). In the study by Mur et al. (Mur et al. 2013), it was demonstrated that throughout the entire duration of ASFV infection (except in the early stage of infection), ASFV antibodies can be detected in oral fluid samples from pigs. As a result, detection methods targeting ASFV antibodies are very important. The commonly used diagnostic methods for detection of ASFV antibody in laboratory include indirect fluorescent antibody test (Carmina et al. 2022), and immunoblotting (Kazakova et al. 2017). Currently, due to its high sensitivity and relatively simple operation, ELISA is recommended by the World Organisation for Animal Health (WOAH) and has become the most suitable and commonly used method for detecting ASFV in large volumes of serum (Qiu et al. 2021). It comprises various formats, such as indirect ELISA (Lv et al. 2021; Pastor et al. 1990; Tabares et al. 1981; Yang et al. 2022), blocking (competitive) ELISA (Caixia et al. 2022; Gao et al. 2022), sandwich ELISA (Vidal et al. 1997; Wang et al. 2019, 2023), and multiplex ELISA. However, it has several shortcomings, such as its complicated operation and time requirement (Gaudreault et al. 2020), so it does not satisfy the needs of large-scale screening and high-throughput analyses. Consequently, a rapid, low-cost, simple method suitable for the high-throughput screening of ASFV p72-specific antibodies is urgently required.

The chemiluminescence immunoassay (CLIA) has been used in veterinary medicine because of its high sensitivity, strong specificity, and simple and rapid application, and because it is not disturbed by stray background light (Liu et al. 2021b, 2017). Here, we used functionalized magnetic particles (MPs) as a new solid-phase carrier material for CLIA. Compared with the traditional CLIA based on a polystyrene microplate, our assay has a larger specific surface area and can be more readily combined with coupled antigens, antibodies, or other bioactive substances. Furthermore, given the stable physical and chemical properties of MPs, they are easily and evenly dispersed in the base solution, which allows a sufficiently strong immune reaction. The assay can also simultaneously detect multiple pathogens with an automatic chemiluminescence detector. At present, CLIA based on magnetic particles (MPCLIA) has been widely used in the clinical and research fields of human medicine, including the diagnosis of infectious diseases (Xu et al. 2022), the detection of cancer biomarkers (Fu et al. 2018), and drug monitoring (Liao et al. 2021). Here, we introduce it for the detection of ASFV infections, and compare this new detection method with the traditional plate-type CLIA.

In this study, we developed two new CLIA methods for detecting antibodies directed against ASFV p72 based on the intact trimeric structure of p72 (the antigen) and a monoclonal antibody (mAb) directed against p72 (mAb-2B8D7; the detection antibody): a traditional plate-type blocking CLIA (p72-CLIA) and an automatic tubular competitive CLIA using magnetic particles (p72-MPCLIA). We evaluated the diagnostic performance of the two methods. Our results show that the higher sensitivity, shorter detection time, and full automation of p72-MPCLIA make it an excellent new tool for the effective prevention and control of ASF on pig farms.

## Materials and methods

### Serum samples

ASFV-negative samples: 432 pig serum samples were collected before the outbreak of ASF in China and tested negative for ASFV using a commercial kit (Lijian Biotechnology, Qingdao, China). These samples were utilized to determine cut-off values, diagnostic specificity (Dsp), and coincidence rates for p72-CLIA, p72-MPCLIA, and a commercial kit.

ASFV-positive samples: 68 pig serum samples were obtained from infected domestic pigs in the field in 2018 and tested positive for ASFV with the same commercial kit (Lijian Biotechnology, Qingdao, China). These samples were used to establish cut-off values, diagnostic sensitivity (Dsn), and coincidence rates for p72-CLIA, p72-MPCLIA, and the commercial kit.

Control serum: The standard positive serum was purchased from the China Institute of Veterinary Drug Control. The standard negative serum (P734) was collected from a clinically healthy pig before the outbreak of ASF, and was determined to be ASFV-negative using a commercial kit (percentage inhibition [PI] = 1%).

### Antibodies, cells, and chemicals

Monoclonal antibody mAb-2B8D7, which detects an epitope of protein p72, was obtained in our previous study (Miao et al. 2023). ExpiCHO-S cells are maintained in our laboratory. Magnosphere™ MS300/Carboxyl magnetic beads were purchased from JSR Corp. (Tokyo, Japan), and alkaline phosphatase (ALP) was purchased from Shanghai Mingjie Biological Company. 1-Ethyl-3-(3-dimethylaminopropyl)-carbohydrate (EDC) and *N*-hydroxysuccinimide (NHS) were purchased from Thermo Fisher Scientific (Waltham, MA, USA).

### Expression and purification of p72 protein

The ASFV gene sequences encoding p72 (B464L) and pB602L (B602L) were accessed from the GenBank database (GenBank MK333180.1) and optimized for synthesis (Optimized Sequences in Supplemental Material). These sequences were cloned into the pcDNA3.1( +) vector. The p72 protein produced by the recombinant plasmid included an N-terminal 6 × His tag. ExpiCHO-S cells (200 mL) were transfected with both recombinant plasmids using the ExpiFectamine™ CHO Transfection Kit (Thermo Fisher Scientific) and incubated at 37 °C with 8% CO2 and continuous shaking. After 18–22 h post-transfection, ExpiFectamine™ CHO enhancer (Thermo Fisher Scientific) was introduced to the culture bottle, which was returned to the incubator and shaken for 8–10 days. The flask contents were centrifuged at 1000 rpm for 10 min and the cell precipitate retained. The precipitate was chilled on ice for 20 min and then treated ultrasonically for 10 min. The product was centrifuged at 10,000 rpm for 10 min and the supernatant collected. Western blotting with an anti-His-tag antibody (Abcam, Cambridge, UK) confirmed the target protein expression. The protein-containing supernatant was further purified using nickel–nitrilotriacetic acid (Ni–NTA) metal affinity chromatography (GE Healthcare, Sweden), and the purified protein’s quality was analyzed by SDS-PAGE.

### Development of p72-CLIA

Checkerboard titration was employed to optimize coating antigen concentration, serum dilution, and enzyme-labeled mAb concentration. Briefly, recombinant p72 protein was coated onto a 96-well chemiluminescence plate (Thermo Fisher Scientific) at 0.5, 1, or 2 μg/mL concentrations in CBS and incubated overnight at 4 °C. The protein was blocked with 3% BSA at 37 °C for 2 h. After three washes with PBST, standard ASF serum (dilutions 1:5, 1:10, 1:20, or 1:40) was added to the plate and incubated at 37 °C for 1 h. Diluted horseradish peroxidase (HRP)-labeled mAb-2B8D7 (0.25, 0.5, or 1 μg/mL) was added and incubated at 37 °C for 30 min. Following three washes with PBST, chemiluminescent substrate (50 μL luminol and 50 μL luminous enhancer) (Key-Bio Biotech Co., Ltd, Beijing, China) was added and incubated at 37 °C for 5 min. Chemiluminescence values were measured using a Varioskan Lux Multimode Microplate Reader (Thermo Fisher Scientific). The optimal recombinant p72 protein concentration, serum dilution, and mAb-2B8D7-HRP concentration were determined based on the blocking rate: PI (%) = (1 – [standard positive CLIA/standard negative CLIA value]) × 100%. Lastly, the two-step reaction time was optimized using the optimal coating antigen concentration, serum dilution, and mAb dilution.

### Development of p72-MPCLIA

Antigen-coupled carboxyl magnetic beads: Magnetic beads (1 mg) were washed three times with 4-morpholine ethanesulfonic acid (MES) solution (pH 5.0) (Aladdin, Shanghai, China) and the volume adjusted to 100 μL (1 mg/100 μL). EDC and NHS were weighed and the concentrations adjusted to 10 mg/mL with MES. EDC (10 μL) and NHS (10 μL) were added to the magnetic beads, mixed, and the beads activated on a mixer for 30 min. The activated magnetic beads were washed three times with MES, and the volume was adjusted to 100 μL (1 mg/100 μL). ASFV p72 protein (2, 4, or 8 μg) was added and the solution mixed thoroughly at room temperature for 3 h. The protein-coupled magnetic beads were washed with MES and the volume kept constant at 100 μL. BSA (10%, 10 μL) was then added and the mixture was shaken at room temperature for 3 h. The magnetic beads were washed three times, diluted to 8, 4, or 2 μg/mg, and stored at 4 °C for later use.

Preparation of mAb-2B8D7–ALP: NaIO_4_ and ethylene glycol were used to activate ALP. The activated ALP was mixed with 1 mg of mAb-2B8D7 and added to 500 mL of 0.1 M CB solution (0.1 M Na_2_CO_3_**,** 0.1 M NaHCO_3_) for overnight coupling. NaBH_4_ was then added for reduction. Finally, the solution was purified with saturated ammonium sulfate precipitation to obtain the ALP-labeled antibody (mAb-2B8D7–ALP), which was stored at − 20 °C for later use (Table 1).

**Table 1:** Parameter settings for the chemiluminescence analyzer

Project	Parameter
Serum sample	50 μL
Magnetic particles coupled antigen	25 μL
2B8D7-ALP	50 μL
Reaction time	10 min
Washing times	4 times
Substrate solution	100 μL
Substrate reaction time	5 min

### Parameter settings for the automatic CLIA analyzer

**Fig. 1:**
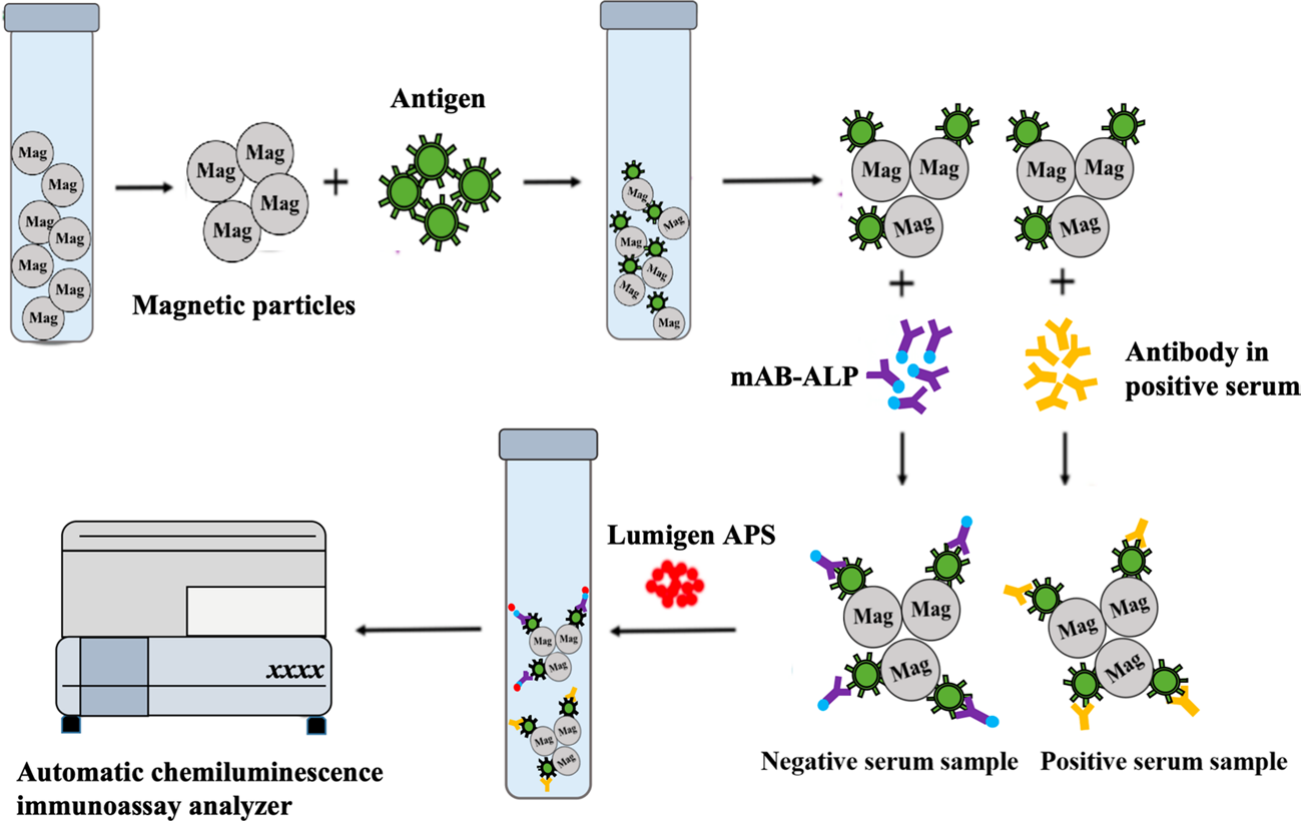
Schematic diagram of competitive MPCLIA reaction steps

The specific reaction steps are shown in Fig. 1.

Optimization of the detection conditions: Different antigen concentrations (2, 4, or 8 μg/mg) were used to test the standard positive and negative sera, according to the experimental processes described above. Detection was repeated three times and the average values calculated. The optimal antigen concentration was selected based on the PI values of the negative and positive sera. The standard negative and positive sera were diluted 1:5, 1:10, 1:20, and 1:40, and mAb-2B8D7–ALP was diluted 1:500, 1:1000, 1:2000, and 1:4000. The best serum dilution and mAb-2B8D7–ALP dilution were selected according to the test results.

The MedCalc software was used for the receiver operating characteristic (ROC) curve analysis to determine the cut-off value, Dsp, and Dsn of the assays. To ensure the validity of the tests, the following conditions were met: chemiluminescence value of the standard negative serum ≥ 5,000,000; PI of the standard positive serum h95%.

### Cut-off value, Dsn, and Dsp

The ROC curve was analyzed with the MedCalc software. The ASF-positive sera (68 samples) and ASF-negative sera (432 samples) with clear known backgrounds were detected under the optimized reaction conditions described above. The 432 ASFV-negative serum samples were used to determine the cut-off value and Dsp of the assay and the 68 ASFV-positive serum samples were used to determine the cut-off value and Dsn of the assay.

### Analytical sensitivity (Ase), repeatability, and cross-reactivity

Ase: The standard ASFV-positive serum was continuously diluted two-fold from 1:2–1:4096, and the PI value was determined simultaneously for p72-CLIA, MPCLIA, and the commercial blocking kit. When the PI value exceeded the cut-off value, the highest dilution of the serum was deemed to be the analytical sensitivity of detection.

Repeatability: To determine the repeatability of the assay within and between batches of samples, six serum samples with different PI values were evaluated in three replicate experiments at different times, using antigens coated in the same and different batches. The coefficient of variation (CV) was calculated based on the chemiluminescence value (CL), as follows:

$$\mathrm{CV}=\frac{\mathrm{Standard}\;\mathrm{deviation}\;(\mathrm{SD})}{\mathrm{Average}\;\mathrm{CL}\;\mathrm{values}}\times100\%$$

Cross-reactivity: To analyze the specificity of the assays, p72-CLIA and p72-MPCLIA were used to test seven kinds of sera containing other swine viruses: porcine circovirus type 2 (PCV2), porcine parvovirus (PPV), foot-and-mouth disease virus (FMDV), porcine reproductive and respiratory syndrome virus (PRRSV), porcine deltacorona virus (PDCoV), classical swine fever virus (CSFV), and porcine epidemic diarrhea virus (PEDV).

### Comparison of coincidence rates

The coincidence rates of the three detection methods (p72-CLIA, p72-MPCLIA, and a commercial blocking kit) were determined using 432 ASFV-negative serum samples collected before the outbreak of ASF in China and 68 ASFV-positive serum samples from infected pigs, collected in the field.

## Results

### Expression and characterization of recombinant p72

**Fig. 2:**
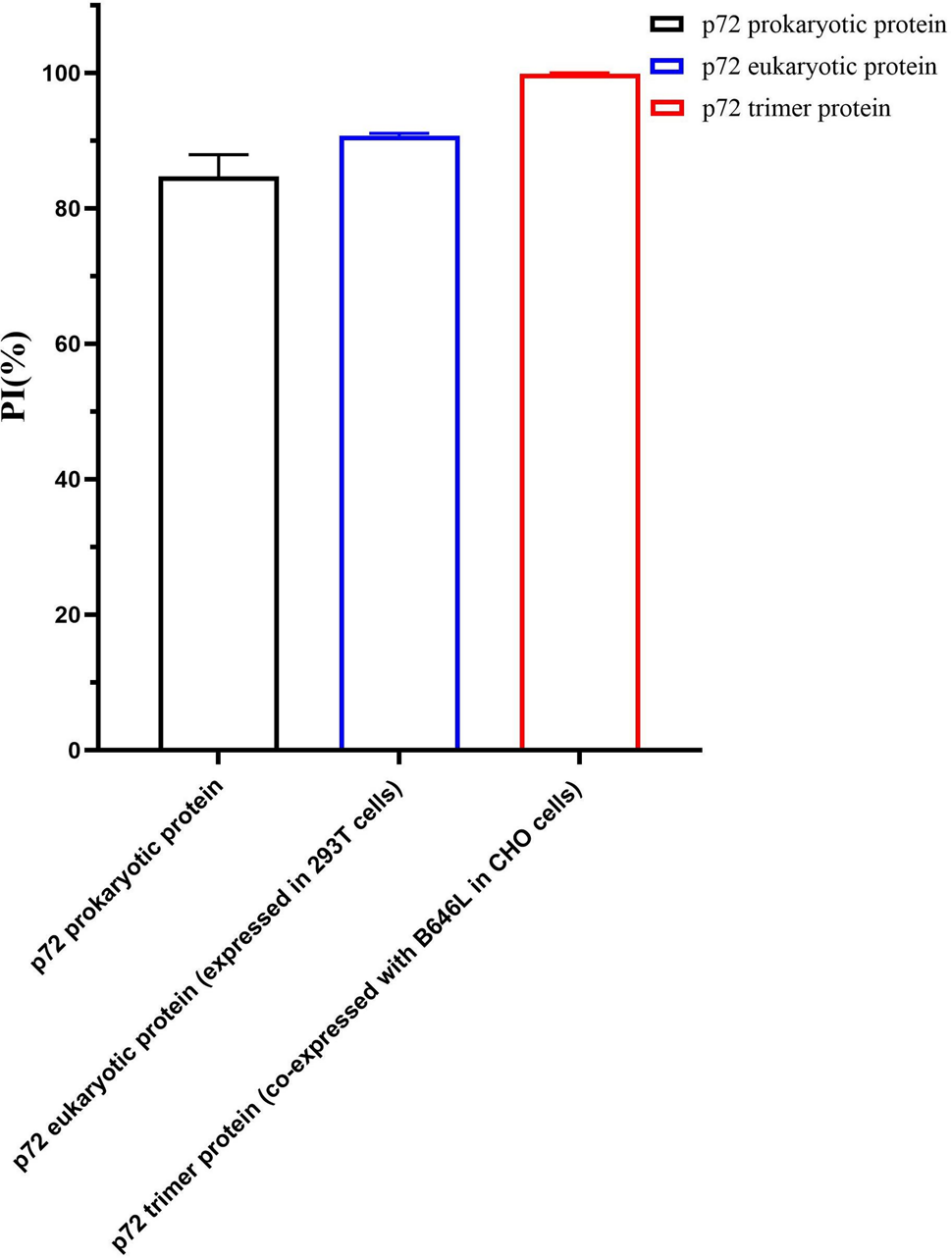
Expression and purification of recombinant p72 protein. (A) The expression of recombinant p72 protein was confirmed with an anti-His-tag antibody. (B) SDS-PAGE confirmed the purity of the recombinant p72 protein. (C) Native PAGE analysis of purified p72. (D) Reactivity of recombinant p72 protein expressed in *Escherichia coli*, 293 T cells or ExpiCHO-S cells was confirmed with ELISA. Plates were coated overnight with the same concentration (0.25 μg/mL) of recombinant protein expressed in *Escherichia coli,* 293 T cells or ExpiCHO-S cells. Standard ASFV-negative and ASFV-positive sera were diluted 1:10 and added to the plates. After incubation for 30 min, mAb-2B8D7–HRP was added. Finally, the blocking rate (PI (%)was calculated

To obtain the correctly folded and assembled p72 trimer, we coexpressed ASFV p72 and pB602L in ExpiCHO-S cells (Liu et al. 2019). Western blots probed with an anti-His antibody showed that the apparent molecular weight of the recombinant p72 protein was ~ 72 kDa, consistent with expectation (Fig. 2A). The recombinant protein was purified with Ni –NTA affinity chromatography, and the purity of the eluted protein was confirmed with SDS-PAGE (Fig. 2B). The trimeric form of the p72 protein was further verified with native PAGE (Fig. 2C), and was consistent with a previous report (Geng et al. 2022). A blocking ELISA was used to compare the detection ability of p72 protein expressed in *Escherichia coli*, 293 T cells or ExpiCHO-S cells (Fig. 2D). The results showed that the recombinant p72 trimer protein with a complete structure and folded in the mammalian expression system performed better diagnostically, making it most suitable for the detection of ASFV antibodies.

### Optimization of p72-CLIA

The optimal reaction conditions were determined based on the results of checkerboard titration and the signal-to-noise ratios. The optimal antigen coating concentration was 1 μg/mL; the optimal serum dilution was 1:5; the optimal enzyme-labeled monoclonal antibody mAb-2B8D7–HRP) concentration was 0.5 μg/mL; and the optimal two-step reaction time was 20 min + 20 min (Fig. S1 in the Supplemental Material).

### Optimization of p72-MPCLIA

Considering the signal-to-noise ratio and economic factors, the optimal concentration of antigen-coupled carboxyl magnetic beads was 4 μg/mg; the optimal serum dilution was 1:5; the optimal dilutions of enzyme-labeled monoclonal antibody mAb-2B8D7–ALP were 1:500, 1:1000 and 1:2000; the optimal reaction time was 10 min; and the optimal blocking solution for competitive MPCLIA was 2% BSA (Fig. S2 in the Supplemental Material).

### Cut-off values, Dsn, and Dsp for p72-CLIA and p72-MPCLIA

**Fig. 3:**
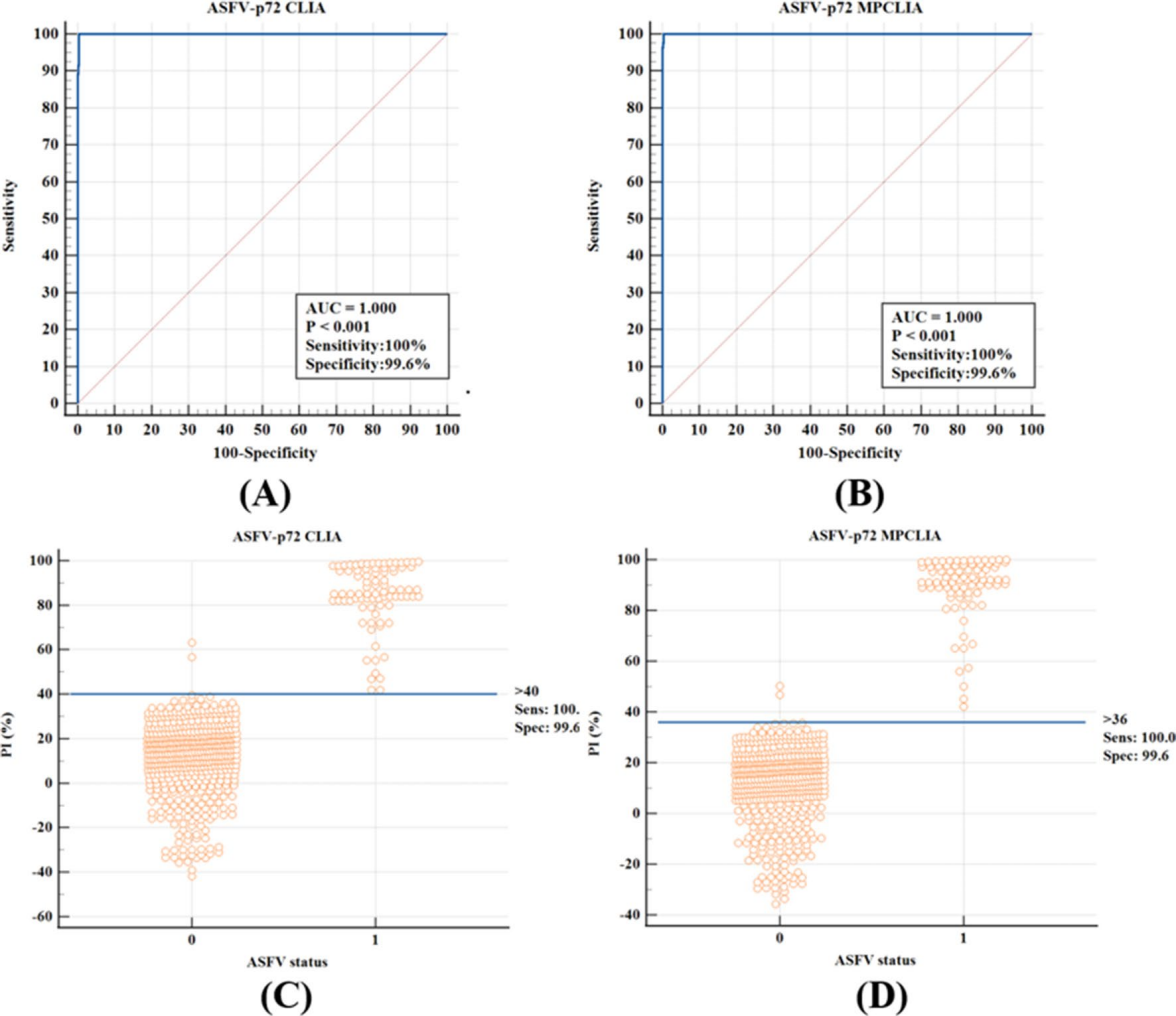
Determination of the cut-off values of p72-CLIA and p72-MPCLIA. (A) and (B) ROC curves of p72-CLIA and p72-MPCLIA, respectively. (C) and (D) Interactive dot diagrams of p72-CLIA and p72-MPCLIA, respectively; 0 represents an ASFV-negative serum sample and 1 represents an ASFV-positive serum sample

Four hundred thirty-two ASFV-negative pig sera and 68 ASFV-positive pig sera with clear backgrounds were tested under the optimized conditions described above, and the cut-off values, Dsn, and Dsp were determined with a ROC curve analysis and background interaction point diagrams. The diagnostic performance of p72-CLIA was compared with that of p72-MPCLIA. The results showed that when the cut-off value of p72-CLIA was 40%, Dsn was 100% and Dsp was 99.6% (Fig. 3A and C). When the cut-off value of p72-MPCLIA was 36%, Dsn was 100% and Dsp was 99.6% (Fig. 3B and D).

### Comparison of Ase of p72-CLIA, p72-MPCLIA, and a commercial ELISA

**Fig. 4:**
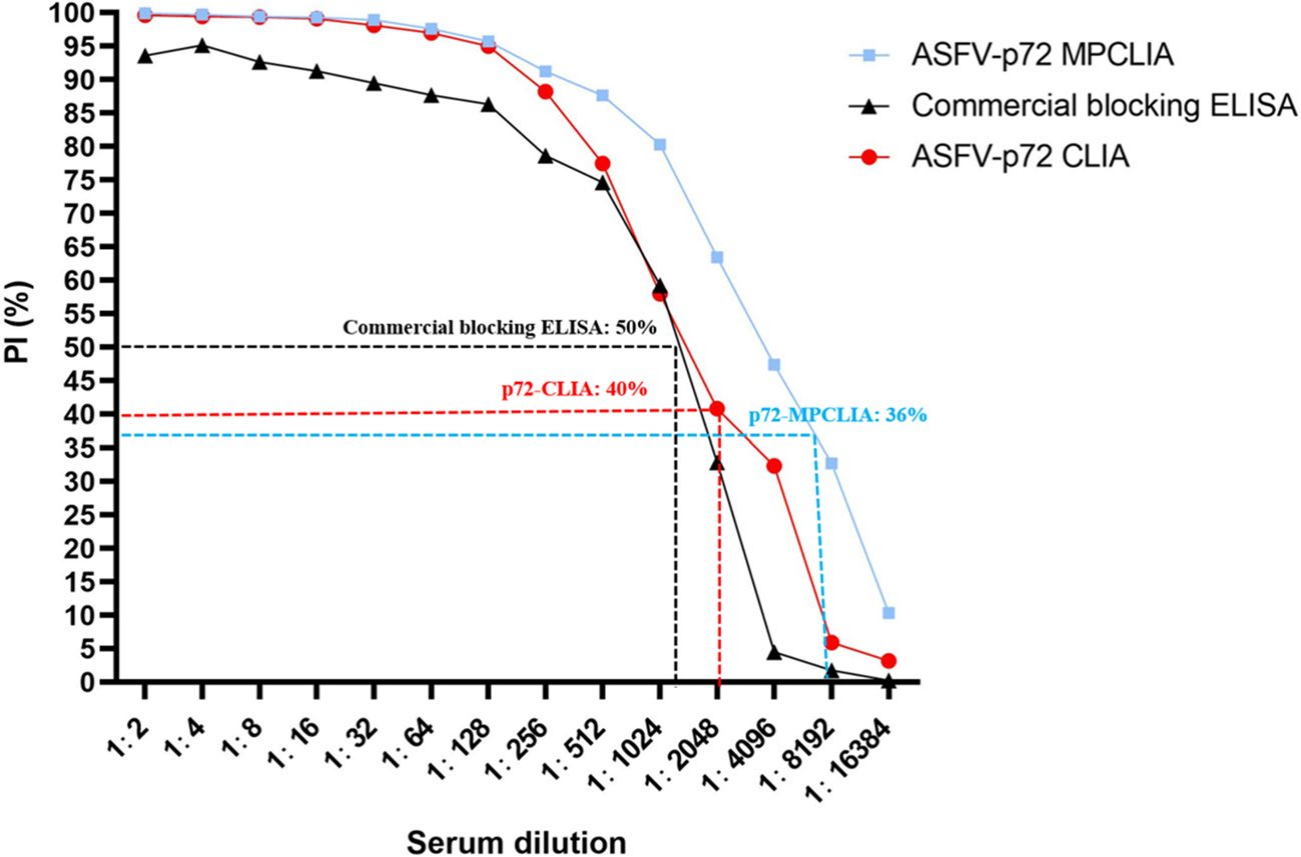
Comparative analytical sensitivities of p72-CLIA, p72-MPCLIA, and commercial ELISA

The analytical sensitivities of p72-CLIA and p72-MPCLIA were determined as twice the continuous dilution of the standard ASFV-positive pig serum, and were compared with that of a commercial blocking kit (Fig. 4). The results showed that the detection limit of p72-CLIA was 1: 2048, the detection limit of p72-MPCLIA was 1:4096, and the detection limit of the commercial blocking ELISA was 1:1024. These data indicate that p72-MPCLIA was more sensitive than both p72-CLIA and the commercial ELISA.

### Repeatability and cross-reactivity of p72-CLIA and p72-MPCLIA

When six pig sera were tested, the intrabatch CV of p72-CLIA was 1.18–14.14% and the interbatch CV was 3.62–14.02% (Table 2). The intrabatch CV of p72-MPCLIA was 2.75–8.51% and the interbatch CV was 2.69–9.78% (Table 3). These results show that the repeatability of p72-MPCLIA was better than that of p72-CLIA.

**Table 2:** Intrabatch and interbatch repeatability of p72-CLIA

Serum sample	Interrun reproducibility			Intrarun reproducibility		
	Mean	SD	CV (%)	Mean	SD	CV (%)
1	3.35	0.46	13.73	3.82	0.54	14.14
2	3.84	0.53	13.80	4.87	0.59	12.11
3	18.56	0.84	4.53	11.05	0.79	7.15
4	15.92	1.78	11.18	10.44	1.02	9.77
5	98.52	3.57	3.62	97.45	1.15	1.18
6	23.33	3.27	14.02	27.18	0.80	2.94

**Table 3:** Intrabatch and interbatch repeatability of p72-MPCLIA

Serum sample	Interrun reproducibility			Intrarun reproducibility		
	Mean	SD	CV (%)	Mean	SD	CV (%)
1	4.41	0.32	7.26	4.88	0.28	5.73
2	2.62	0.17	6.49	2.89	0.20	6.92
3	12.78	0.89	6.96	13.93	0.91	6.53
4	13.01	1.01	7.76	13.32	1.04	7.80
5	99.38	2.68	2.69	99.72	2.75	2.75
6	20.13	1.97	9.78	22.08	1.88	8.51

**Fig. 5:**
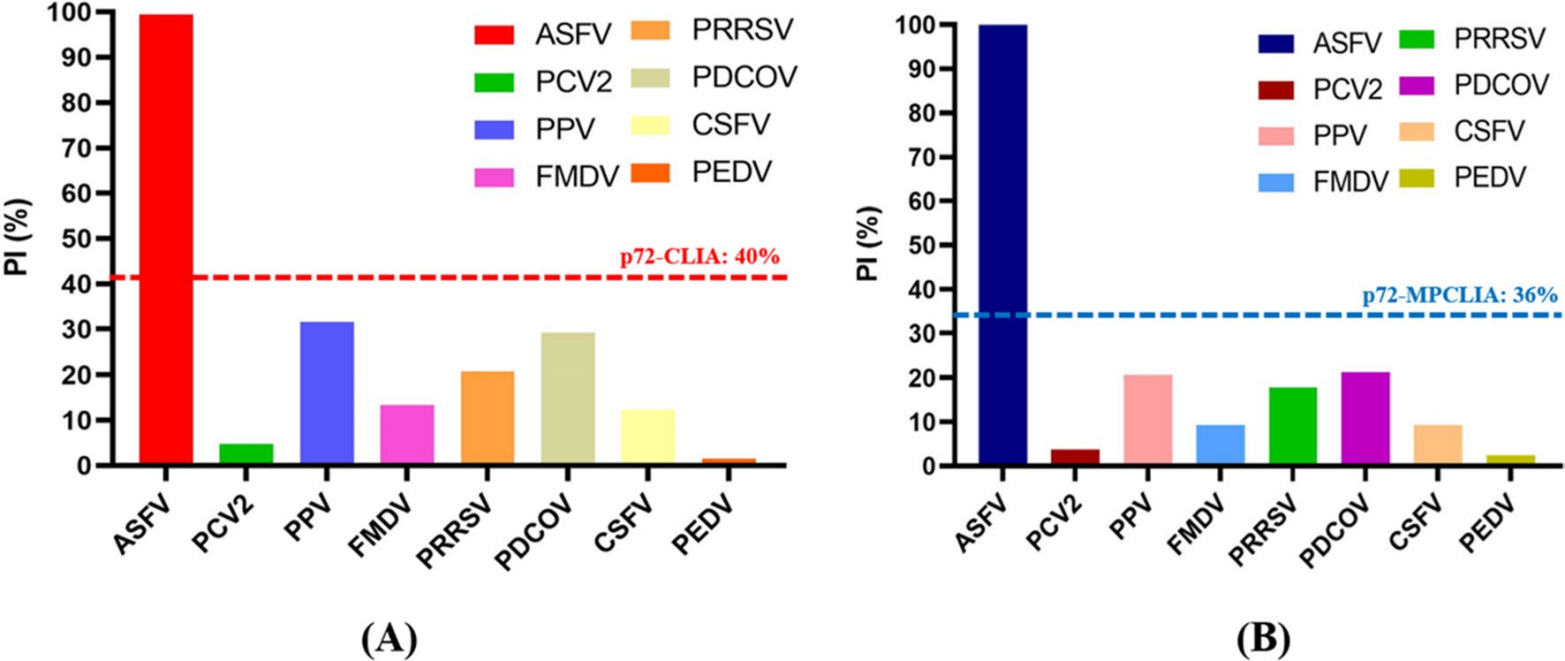
Specificities of p72-CLIA and MPCLIA. (A) PI values for eight porcine viruses tested with p72-CLIA. (B) PI values for eight porcine viruses tested with p72-MPCLIA

To evaluate the specificity of p72-CLIA and p72-MPCLIA, we tested their reactivity with seven different porcine viruses (PCV2, PPV, FMDV, PRRSV, PDCoV, CSFV, and PEDV). The results for p72-CLIA (Fig. 5A) and p72-MPCLIA (Fig. 5B) showed that the PI values for nonspecific positive serum were significantly lower than the cut-off values, indicating that the p72-CLIA and p72-MPCLIA developed here did not cross-react with these virus-infected sera, and therefore showed good specificity.

### Coincidence rates

The coincidence rates of p72-CLIA, p72-MPCLIA, and the commercial Lijian ASFV-Ab ELISA were determined by testing 500 pig serum samples with clear backgrounds. The results (Table 4) showed that the coincidence rates of p72-CLIA and p72-MPCLIA with the commercial kit in detecting ASFV-positive and ASFV-negative sera were the same (100% and 99.53%, respectively).

**Table 4:** Coincidence rates of p72-CLIA, p72-MPCLIA, and commercial ELSIA

Sample source	Total no. of samples	p72-CLIA			p72-MPCLIA			Commercial blocking kit (Lijian)		
		No. of samples positive	No. of samples negative	Coincidence rate (%)	No. of samples positive	No. of samples negative	Coincidence rate (%)	No. of samples positive	No. of samples negative	Coincidence rate (%)
Infection positive pig serum	68	68	0	100%	68	0	100%	68	0	100%
Negative pig serum	432	2	430	99.53%	2	430	99.53%	2	430	99.53%

## Discussion

As the world’s largest producer and consumer of pigs, China has experienced immeasurable economic losses from ASF, and ASF still poses a serious threat to the pig and related industries (Zhang et al. 2023). At present, the prevention and control of ASF mainly depends on its early accurate diagnosis and strict biological control. Therefore, it is important to develop a fast, accurate, sensitive, and simple laboratory detection technology for the prevention and control of ASF. With the recent appearance of naturally attenuated ASFV strains that do not cause death in infected pigs, it is difficult to investigate the epidemic status of ASF on pig farms in a timely manner unless ASFV nucleic acids or antigens are monitored regularly. Because antibodies are generated in infected pigs, infected pigs can be effectively identified by the detection of ASFV-directed antibodies. Therefore, a method of detecting ASFV-specific antibodies is essential for the diagnosis of ASFV infections.

ASFV p72 exists as a trimeric protein in the capsid of ASFV (Liu et al. 2019), and only when p72 and pB602L are co-expressed is the complete structure of p72 correctly folded and assembled (Cobbold et al. 2001; Epifano et al. 2006). p72, with a complete and natural structure, can be used to accurately distinguish ASFV-positive and -negative sera and reduce false positive reactions during antibody detection (Cubillos et al. 2013). In a study by (Geng et al. 2022), the correctly folded p72 trimer with its natural structure was obtained by co-expressing p72 and pB602L in a eukaryotic expression system. A colloidal gold immunochromatographic test strip was then developed that showed good diagnostic performance. In a study by Zhu et al. (Zhu et al. 2021), acid-treated trimeric p72 (with a deformed structure) was used as the capture antigen and marker antigen to develop colloidal gold strips, which had a sensitivity of only 1:1000. Therefore, in the present study, we co-expressed p72 with the molecular chaperone pB602L in a mammalian expression system to obtain the natural trimeric form of p72 with a complete structure. In our research, the mAb-2B8D7 was prepared from mice immunized with the p72 protein expressed in *E. coli*. However, the blocking rate of the standard ASFV-positive serum was relatively low (PI = 85%) when the plate was coated by *E. coli*-expressed p72 protein. Interestingly, the blocking rate slightly improved (PI = 90.5%) when using p72 protein expressed in 293 T cells. Notably, the blocking rate was remarkably high (PI = 99.8%) when using the trimer protein (natural state) co-expressed with B646L and p72 in ExpiCHO-S cells. This difference in blocking rates may be attributed to the misfolding of p72 proteins (unnatural state) expressed in *E. coli* and 293 T cells, which potentially affect the blocking ability of p72 mAbs. This phenomenon has been observed, but we do not yet know the specific reasons.

The advantages of CLIA include high sensitivity, strong specificity, simplicity, rapidity, and easy automation, and it needs no external light source and is not disturbed by stray background light. This method has been widely used in veterinary research, including in the detection of FMDV infections (Liu et al. 2021c, 2017, 2018). Therefore, we developed a traditional chemiluminescence method (p72-CLIA) and a magnetic particle tube chemiluminescence method (p72-MPCLIA) based on the p72 trimer, and compared the diagnostic performances of these two methods. The results showed that both p72-MPCLIA and p72-CLIA have good Dsn (100%), Dsp (99.6%), and repeatability. p72-MPCLIA and p72-CLIA also showed good diagnostic performances, and their coincidence rate with the commercial ELISA kit were the same (100%). The two false-positive sera identified when testing ASFV-negative serum with clear backgrounds may have been contaminated with bacteria because the sera had been stored for too long, and the bacteria may have expressed endogenous HRP, resulting in a false positive reaction (Matson 2023). Compared to the chemiluminescent magnetic microparticle immunoassay for the detection of antibodies against ASFV developed by Shi et al. (Shi et al. 2023), which is an indirect method based on the p30 recombinant protein, the p72-MPCLIA and p72-CLIA in this study are competitive methods based on p72 mAb-2B8D7, which have higher specificity and contribute to improving the accuracy of detection. Furthermore, the epitope “^281^PENSHNIQTA^290^” identified by mAb-2B8D7 was published in our previous research (Miao et al. 2023) and exhibits high conservation across 27 isolates from nine distinct ASFV genotypes (genotype I, II, III, IV, V, VIII, IX, X, XX). Thus, the p72-MPCLIA and p72-CLIA can be used to detect antibodies against ASFV genotype I and II that are epidemic in China, although only ASFV genotype II-positive serum samples collected in the early days of the outbreaks were detected in this study.

The detection time is an important index with which to evaluate the diagnostic ability of an assay, and its length determines the efficiency of a detection method. In geographic regions with strong likelihood of ASFV infection, time-consuming detection methods may affect the prevention and control of ASF. The p72-MPCLIA method established here requires only 15 min to produce a test result, whereas p72-CLIA requires 45 min and commercial ELISA kits, such as the INgezim PPA COMPAC Blocking ELISA Kit (Li et al. 2022a), require 90 min. Therefore, the method we established effectively shortens the detection time and improves the detection efficiency for ASFV-directed antibodies. Furthermore, the diagnostic performance of MPCLIA was not similar to that of other kits in a short time. Moreover, combined with a fully automated chemiluminescence detection instrument could simultaneously detect and screen for diseases with similar clinical symptoms, such as ASF, highly pathogenic PRRS infection, classical swine fever (CSF) and swine erysipelas. Thus, p72-MPCLIA not only provides important material and technical support for the prevention, control, and purification of ASF in China, but also provides a new reference for resolving the complex problem of mixed infections with multiple pathogens faced by the pig industry.

In summary, two CLIAs, p72-CLIA and p72-MPCLIA, were developed in this study based on p72 mAb-2B8D7, and their diagnostic performance was compared. Both methods showed good Dsn, Dsp, and repeatability, but p72-MPCLIA showed greater sensitivity (1:4096) than p72-CLIA. p72-MPCLIA detects antibodies directed against ASFV p72 highly efficiently in only 15 min, more quickly and accurately than the p72-CLIA method or traditional ELISAs. In summary, the p72-MPCLIA antibody detection method developed in this study is expected to be a valuable tool for the large-scale screening of pigs for ASFV and thus for controlling the spread of ASFV.

## Supplementary Information

Below is the link to the electronic supplementary material.

Supplementary file1 (DOCX 482 KB)

## Data Availability

Data and materials are available from the corresponding authors upon reasonable request.
