# Supplementary material for: Development of plate-type and tubular chemiluminescence immunoassay against African swine fever virus p72
Source: Appl Microbiol Biotechnol. 2024 Aug 2;108:431. doi: 10.1007/s00253-024-13249-5 (PMC11297061; doi:10.1007/s00253-024-13249-5)
Supplement: Supplementary file 1 [file 253_2024_13249_MOESM1_ESM.docx]

**Applied Microbiology and Biotechnology**

**Development of plate-type and tubular chemiluminescence immunoassay against African swine fever virus p72**

Chun Miao^1,2^, Junjun Shao^1,2*^, Sicheng Yang^1,2^, Shenghui Wen^1,2^, Yunyun Ma^1,2^, Shandian Gao^1,2^, Huiyun Chang^1,2^, Wei Liu^1,2*^

^1^State Key Laboratory for Animal Disease Control and Prevention, Lanzhou Veterinary Research Institute, Chinese Academy of Agricultural Sciences, Lanzhou, China.

^2^Gansu Province Research Center for Basic Disciplines of Pathogen Biology, Lanzhou Veterinary Research Institute Chinese Academy of Agricultural Sciences, Lanzhou, China.

*** Correspondence:** Junjun Shao, [shaojunjun@caas.cn](mailto:shaojunjun@caas.cn)**;** Wei Liu, liuwei10@caas.cn**.**

**Optimized sequence of the p72 gene**

5’-ATGGCTTCAGGGGGAGCATTTTGTCTAATAGCGAATGATGGTAAGGCAGATAAGATCATCCTTGCCCAAGACCTGTTGAATAGCCGTATCTCTAACATCAAGAACGTCAATAAAAGCTATGGCAAACCGGACCCGGAACCGACGCTGTCCCAAATTGAGGAAACCCATCTGGTGCATTTTAATGCGCACTTCAAACCGTATGTTCCGGTGGGCTTTGAATACAATAAGGTGCGCCCACACACCGGCACTCCGACGCTGGGTAATAAGTTGACCTTCGGCATTCCGCAATATGGTGACTTTTTCCACGACATGGTTGGCCACCATATTCTGGGTGCATGTCATAGCAGCTGGCAGGACGCGCCCATCCAAGGTACGAGCCAAATGGGTGCGCACGGCCAGCTGCAGACCTTTCCGCGTAACGGCTACGACTGGGATAATCAGACCCCGTTGGAAGGTGCGGTGTACACCCTGGTCGATCCGTTTGGCCGTCCGATTGTTCCGGGTACCAAAAACGCGTATCGTAACCTCGTCTATTACTGCGAATACCCGGGCGAGCGCCTGTACGAAAACGTTCGTTTTGATGTGAATGGCAACTCTCTGGATGAGTATTCCAGCGATGTTACCACCCTCGTGCGTAAATTCTGTATCCCGGGCGATAAAATGACCGGTTATAAACATCTGGTCGGTCAGGAGGTGTCCGTTGAAGGCACCTCTGGTCCGCTGTTGTGCAACATCCACGATCTGCATAAGCCGCATCAATCCAAACCGATCCTAACTGACGAGAACGACACCCAGCGTACGTGCAGCCATACCAACCCGAAGTTTCTGAGCCAGCACTTTCCGGAAAACAGCCACAACATCCAGACCGCTGGTAAACAGGACATCACCCCGATTACCGACGCAACCTACCTGGACATTCGTCGTAACGTGCACTACTCCTGTAATGGCCCTCAGACCCCGAAGTATTATCAGCCACCGCTGGCTCTCTGGATTAAGCTGCGCTTCTGGTTCAACGAGAACGTCAACCTGGCGATCCCGTCCGTTTCTATCCCGTTTGGTGAACGTTTCATCACCATTAAGCTGGCAAGCCAAAAAGATCTTGTAAATGAGTTTCCTGGTCTGTTCGTTCGCCAGAGCAGATTTATCGCTGGTCGTCCAAGTCGCAGAAACATTCGCTTCAAGCCGTGGTTTATCCCAGGAGTTATTAACGAGATCTCGTTGACCAACAACGAGCTGTACATAAACAACCTGTTTGTTACCCCGGAGATTCACAATCTGTTCGTGAAACGTGTTCGTTTCAGCCTGATCCGCGTGCACAAGACTCAAGTTACCCATACTAATAATAACCACCACGACGAGAAACTGATGTCAGCTTTGAAGTGGCCGATCGAATACATGTTTATAGGGCTGAAACCAACCTGGAATATCTCCGACCAGAACCCGCACCAGCATCGTGATTGGCATAAATTCGGTCATGTTGTGAACGCCATTATGCAACCGACACATCACGCGGAAATCTCTTTTCAGGATAGAGACACCGCCTTACCGGACGCGTGCAGCAGCATTTCTGATATCTCTCCGGTGACGTACCCGATTACTCTGCCGATCATTAAGAACATCTCCGTTACCGCGCACGGCATCAACCTGATTGACAAGTTCCCGAGCAAATTCTGCAGCTCGTACATTCCGTTCCACTATGGTGGTAACGCCATCAAGACCCCGGATGATCCTGGCGCAATGATGATTACGTTTGCACTGAAACCGCGTGAGGAGTACCAACCGAGCGGTCACATCAACGTTTCACGCGCGCGTGAGTTCTATATCAGTTGGGACACGGACTACGTGGGTAGCATTACTACGGCCGACTTGGTCGTGAGCGCGTCCGCGATCAATTTCTTGTTGTTGCAAAACGGCAGCGCTGTTCTGCGTTATAGCACCTGA-3’

**Optimized sequence of the B646L gene**

5’-ATGAGGCTGAGCGTGTGTCTGCTGCTGCTGACTCTGGCTCTGTGTTGCTACAGAGCTAATGCTGAATTCATGGCTGAGTTCAATATCGACGAGCTCCTGAAGAACGTGCTGGAGGACCCTTCCACCGAGATCTCTGAGGAGACCCTGAAGCAGCTGTACCAGCGAACAAACCCTTACAAGCAGTTTAAGAACGATAGCAGAGTGGCCTTTTGTAGCTTCACCAACCTCAGGGAGCAGTACATCAGAAGGCTGATCATGACAAGCTTCATCGGCTACGTGTTTAAGGCTCTGCAGGAGTGGATGCCTAGCTACTCAAAGCCTACCCACACCACCAAGACCCTGCTGTCCGAGCTGATCACACTTGTGGATACACTGAAGCAGGAGACAAACGACGTGCCTTCTGAAAGCGTGGTGAACACAATTCTGTCTATCGCCGACTCTTGCAAGACACAGACCCAGAAATCCAAGGAGGCCAAGACAACAATCGACAGCTTTCTGAGAGAGCATTTCGTGTTCGATCCCAATCTGCACGCCCAAAGCGCCTACACCTGCGCCGACACCAACGTGGATACCTGTGCCAGTATGTGTGCCGATACCAACGTCGATACCTGTGCCAGTATGTGCGCCGACACCAACGTCGACACCTGCGCCTCAACTTGCACATCGACAGAGTACACCGATCTGGCCGACCCAGAGAGAATCCCTCTCCATATTATGCAAAAGACCTTGAACGTGCCAAACGAGCTGCAGGCCGATATCGACGCTATCACACAGACTCCACAGGGCTACCGAGCCGCCGCTCATATCCTGCAGAACATCGAGCTGCACCAGTCTATTAAGCACATGCTGGAAAACCCTAGGGCCTTTAAGCCTATCCTGTTCAACACAAAGATCACCAGATACCTGTCTCAGCACATCCCTCCTCAGGATACATTCTACAAGTGGAACTATTACATCGAGGATAACTATGAGGAGCTGAGAGCCGCCACCGAGTCTATCTACCCTGAGAAGCCTGATCTGGAGTTTGCCTTTATCATCTATGATGTGGTGGATAGCAGCAACCAGCAGAAGGTGGATGAGTTCTACTACAAGTATAAGGATCAGATCTTTTCTGAAGTGAGCTCCATCCAGCTGGGCAACTGGACACTGCTGGGCTCTTTCAAGGCCAACAGAGAGAGATACAACTACTTTAACCAGAACAACGAGATCATCAAGAGAATCCTGGACAGGCACGAGGAGGATCTGAAGATCGGCAAGGAGATCCTGAGGAATACCATCTACCACAAGAAGGCCAAGAATATCCAGGAAACTGGACCTGATGCCCCTGGCCTGAGCATCTACAACAGTACATTCCACACAGATTCTGGCATCAAGGGCCTGCTGAGCTTTAAGGAGCTGAAGAACCTGGAAAAGGCTAGCGGCAACATTAAGAAGGCTAGAGAGTACGATTTCATTGATGATTGTGAGGAGAAGATCAAGCAGCTGCTGTCTAAGGAGAACCTGACCCCTGATGAGGAGTCCGAGCTGATTAAGACCAAGAAGCAGCTCGACAATGCTTTGGAAATGCTCAACGTGCCTGACGATACCATTCGGGTGGATATGTGGGTTAATAATAATAACAAACTCGAAAAGGAGATTCTGTACACAAAGGCCGAGCTCGGAGGATCTTCTGGCGGACACCATCACCACCACCACTGA-3’

**SUPPLEMENTARY FIG 1** Optimum reaction conditions for p72-CLIA. (A) (B) (C) When the concentration of mAb-2B8D7–HRP was 0.25, 0.5, or 1 μg/mL, the coating antigen concentration and serum dilution were optimized by checkerboard titration. N, negative serum sample; P, positive serum sample. The figure shows the PI values for different coating antigen concentrations when the serum was diluted 1:5. (D) Optimization of the reaction time.

**SUPPLEMENTARY FIG 2** Optimum reaction conditions for p72-MPCLIA. (A) When the dilution of mAb-2B8D7–ALP was 1:500, the coating antigen concentration and serum dilution were optimized. N, negative serum sample; P, positive serum sample. The figure shows the PI values for different coating antigen concentrations when the serum was diluted 1:5. (B) The optimum mAb-2B8D7–ALP dilution (1:500, 1:1000, 1:2000, and 1:4000) was optimized at the optimum coating antigen concentration (4 μg/mg) and serum dilution (1:5). (C) Optimization of the reaction time. (D) Assay for screening blocking agents.
